# Dietary supplementation of lignocellulose promotes the growth of Cherry Valley ducks by improving intestinal function

**DOI:** 10.1016/j.vas.2025.100558

**Published:** 2025-12-17

**Authors:** Xinzhi Geng, Zhenzhen Chen, Jian Wang, Biao Dong, Jing Ge, Minmeng Zhao, Long Liu, Daoqing Gong, Haixia Liu, Tuoyu Geng

**Affiliations:** a College of Animal Science and Technology, Yangzhou University, Yangzhou 225009, Jiangsu Province, China; b Department of Animal Science and Technology, Jiangsu Agri-animal Husbandry Vocational College, Taizhou 225300, Jiangsu Province, China; c Joint International Research Laboratory of Agriculture and Agri-Product Safety of Ministry of Education of China, Yangzhou University, Yangzhou 225009, Jiangsu Province, China

**Keywords:** Lignocellulose, Antioxidant capacity, Meat duck, Growth performance, Intestinal morphology

## Abstract

Dietary fiber contributes to improving intestinal morphology, barrier integrity, and microbial balance, thereby enhancing nutrient utilization and growth performance in animals. This study evaluated the effects of supplementing lignocellulose on intestinal function and growth performance in meat ducks. A total of 180 one-day-old Cherry Valley ducks were randomly assigned to the control group (CON), lignocellulose group 1 (LC1), and lignocellulose group 2 (LC2) (6 replicates each group, 10 ducks each replicate). Ducks in the CON group were fed a basal diet; the LC1 group received the basal diet supplemented with 0.6% lignocellulose from days 1 to 42, while the LC2 group received the basal diet supplemented with 0.3 % lignocellulose from days 1 to 21 and 0.6 % from days 22 to 42. Lignocellulose supplementation increased average daily gain in the LC1 group (*P* < 0.05), and improved feed conversion ratio in the LC2 group before day 28 (*P* < 0.05). Additionally, lignocellulose increased intestinal length (*P* < 0.05) and weight (*P* < 0.05), and improved the intestinal structure (*P* < 0.05) and antioxidant capacity (*P* < 0.05). However, the expression of genes associated with tight junctions, immunity, and inflammation was not significantly affected. Overall, dietary lignocellulose promotes growth in meat ducks by improving intestinal morphology and antioxidant capacity without affecting the expression of genes involved in intestinal tight junction and immunity or inflammation. The magnitude of benefits depends on the supplementation level, duration, and the age of birds.

## Highlights

• Appropriate dietary lignocellulose promotes the growth of meat ducks.
• Dietary lignocellulose improves intestinal growth, structure and antioxidant capacity.
• Dietary lignocellulose does not cause intestinal inflammation.
• The effects of dietary lignocellulose depend on its dosage and age of birds.

## 1 Introduction

Duck meat is a high-quality animal protein source rich in unsaturated fatty acids, and China ranks first worldwide in both the production and consumption of duck meat. Previous studies have reported that appropriate use of feed additives such as probiotics, herbs, and cellulose can enhance the performance and health of livestock and poultry by improving intestinal disease resistance, modulating gut microflora, and promoting intestinal development and digestive function (Diaz-Sanchez et al., 2015). Although crude fiber is traditionally considered to impair nutrient digestibility due to its anti-nutritional properties, recent findings indicate that dietary fiber supplementation can improve animal productivity. For example, Iji et al. reported that crude fiber supplementation improved animal performance, and the response was closely related to the source and the dietary inclusion level of fiber (Iji et al., 2001).

Dietry fiber is generally classified as soluble or insoluble. The increase of soluble fiber in animal diets can increase the intestinal viscosity, promote the proliferation of pathogenic bacteria, and induce abdominal distension and intestinal inflammation (Owusu-Asiedu et al., 2006). On the contrary, insoluble fiber possesses strong water-absorption capacity and can stimulate intestinal peristalsis, promote intestinal development, improve intestinal structure and function, and suppress the proliferation of harmful bacteria, thereby enhancing animal production performance. Several studies have shown that supplementing an appropriate amount of dietary fiber to low-fiber diets can improve nutrient utilization and intestinal microbiota composition, ultimately enhancing growth in broilers (González-Alvarado et al., 2007; Jha et al., 2019). Although lignocellulose, as an insoluble crude fiber, can influence intestinal health primarily through its physical properties, its beneficial effects in meat ducks have not been completely understood. Given that the efficacy of dietary fiber depends on its type and dosage (Han et al., 2017), there is a study indicating that appropriate levels of dietary fiber can enhance gizzard development and improve intestinal morphology (Han et al., 2016).

Mechanistically, lignocellulose can be fermented and degraded to produce beneficial metabolites such as short-chain fatty acids, which exert anti-inflammatory effects and thereby improve the intestinal health in poultry (Arpaia & Rudensky, 2014; Zeitz et al., 2019). Moreover, dietary lignocellulose supplementation has been shown to enhance the antioxidant capacity of animals, which is previously demonstrated in the study where antioxidant capacity was increased in the jejunal and ileal mucosa of broilers by dietary lignocellulose (Zeitz et al., 2019). Key indicators of antioxidant activity typically include total superoxide dismutase **(T-SOD**), glutathione (**GSH**), glutathione S-transferase (**GST**), malondialdehyde (**MDA**) and total antioxidant capacity (**T-AOC**). **T-SOD** plays a critical role in maintaining redox balance and protecting cellular integrity by scavenging superoxide radicals. **GSH**, a major non-enzymatic antioxidant, neutralizes reactive intermediates, whereas **GST** catalyzes GSH-mediated conjugation reactions to promote detoxification of oxidative byproducts and xenobiotics . T-AOC reflects the overall body's antioxidant status, while MDA, a significant byproduct of lipid peroxidation, indicates the extent of oxidative damage. Under oxidative stress, reduced SOD and GST activities, decreased GSH levels, and increased MDA concentrations are indicative of aggravated oxidative injury.

Based on the findings from previous studies on lignocellulose in other poultry species, particularly chickens, we hypothesized that supplementing lignocellulose improves production performance in ducks by enhancing intestinal function and microbial composition. Therefore, this study aimed to address this hypothesis by determining the effects of different dietary levels of lignocellulose on intestinal development, production performance, morphological structure, and intestinal antioxidant capacity of Cherry Valley ducks. These results are expected to provide a reference for the application of lignocellulose in meat duck production.

## 2 Materials and methods

### 2.1 Experimental animals, production performance measurement and sample collection

All the animal experiments were conducted in accordance with ethical guidelines and regulations for animal use and welfare. All animal protocols were approved by the Yangzhou University Animal Ethics Committee (approval number SYXK(Su)2021–0026).

A total of 180 one-day-old healthy Cherry Valley male ducks with similar body weight were selected and randomly assigned to three groups (CON, LC1, and LC2), with six replicates per group and ten ducks per replicate. All ducks were raised at the experimental base of the National Waterfowl Gene Bank (Taizhou, Jiangsu, China).

A corn-soybean meal-based mash diet was formulated as the basal diet. It met the nutrient requirements of Cherry Valley ducks according to national standards and the Cherry Valley Duck Management Guide, and contained no antibiotics. The ingredient composition and nutrient levels are presented in Table 1. Lignocellulose used in this study was supplied by Arbocel (Rosenberg, Germany), with the following composition: 7.7 % moisture, 1.0 % crude protein, 65.3 % crude fiber, 0.30 % crude fat, 0.50 % ash, and 25.1 % nitrogen-free extract. The product had an average fiber length of 200 μm and a diameter of 35 μm. Similar to other insoluble fibers such as wheat bran, it exhibits strong water-holding capacity (dependent on particle size), which contributes to improved nutrient utilization and modulation of gut microbiota (Mongeau & Brooks, 2016).

**Table 1:** The composition and nutritional level of the basal diet used in this study (air-dried basis).

Item	1∼21 d of age			22∼42 d of age		
	CON	LC1	LC2	CON	LC1	LC2
Composition, %						
Corn (CP 8 %)	56.50	56.20	56.35	66.75	66.45	66.45
Soybean meal (CP 44.2 %)	37.75	37.45	37.60	27.10	26.80	26.80
Lignocellulose		0.60	0.30		0.60	0.60
Limestone	1.30	1.30	1.30	1.50	1.50	1.50
Soybean oil	1.80	1.80	1.80	2.00	2.00	2.00
Calcium hydrogen phosphate	1.50	1.50	1.50	1.50	1.50	1.50
L-Lysine	0.05	0.05	0.05	0.05	0.05	0.05
DL-Methionine	0.10	0.10	0.10	0.10	0.10	0.10
Premix^①^	1.00	1.00	1.00	1.00	1.00	1.00
Total	100.00	100.00	100.00	100.00	100.00	100.00
Nutritional level^②^						
Metabolic energy, MJ/kg	12.02	11.95	11.98	12.40	12.33	12.33
Crude protein, %	21.21	21.05	21.13	17.32	17.17	17.17
Crude fat, %	4.52	4.50	4.51	4.88	4.86	4.86
Crude fiber, %	3.53	3.89	3.71	3.13	3.50	3.50
Calcium, %	1.04	1.04	1.04	1.08	1.08	1.08
Total phosphorus, %	0.73	0.73	0.73	0.69	0.69	0.69
Available phosphorus, %	0.41	0.41	0.41	0.40	0.40	0.40
Lysine, %	1.19	1.19	1.19	0.93	0.93	0.93
Methionine, %	0.41	0.41	0.41	0.37	0.37	0.37

Note: Each kg of premix contains: Vitamin A 11 000 IU, Vitamin D3 2 400 IU, Vitamin E 24 IU, Vitamin K 2.2 mg, Vitamin B1 1.3 mg, Vitamin B2 7.6 mg, Vitamin B6 2.9 mg, Vitamin B12 0.01 mg, Pantothenic acid 11.0 mg, niacin (Niacin) 55.0 mg, Choline 480.0 mg. biotin 0.04 mg, folic acid 1.8 mg, copper 10.0 mg, zinc 90.0 mg, iron 100.0 mg, manganese 85.0 mg, iodine 0.5 mg, and selenium 0.2 mg. Nutritional level was the calculated value.

The CON group was fed with a basal diet, the LC1 group received a basal diet supplemented with 0.6 % lignocellulose, whereas the LC2 group received the basal diet supplemented with 0.3 % lignocellulose from day 1 to 21 and 0.6 % lignocellulose from day 22 to 42. The selection of lignocellulose supplementation levels (0.3 % and 0.6 %) was based on rigorous literature review and meat duck-specific physiological characteristics. Previous studies indicate that 0.2–0.8 % dietary lignocellulose or insoluble fiber can effectively improve intestinal health and production performance in poultry without inducing anti-nutritional effects (Zeitz et al., 2019; Han et al., 2016). However, supplementation above 0.8 % has been reported to increase intestinal digesta viscosity and reduce nutrient digestibility in young poultry (Owusu-Asiedu et al., 2006). In this study, 0.6 % was chosen as the upper limit to avoid potential digestive burden while maintaining its beneficial effects. All ducks were fed *ad libitum* with free access to clean water. Nipple drinkers and automatic feeders were used to ensure voluntary intake and adequate nutrient supply. Feed intake per replicate was recorded daily, and body weight was measured weekly. Fasting commenced before midnight on the day prior to weighing.

From day 1 to day 21, ten ducks per replicate were raised in plastic cages (1.4 *m*× 0.7 *m*× 0.5 m; length × width × height) and from day 22 to day 42, they were transferred to an elevated plastic-net floor (1.2 m), with 10 ducks per pen. The brooding period was 3 weeks long. The brooding temperature was initially set at 32 °C for the first two days, and then reduced by 0.5 °C per day until reaching the ambient temperature of 25 ± 2 °C on day 15, which was maintained thereafter. The 24-hour lighting period was provided from days 1 to 3, reduced to 23 h from days 4 to 8,and subsequently decreased by 1 hour per day until reaching 16 h of light per day. The relative humidity was 60–70 % for the first week of age, followed by 50–55 % from days 8 to 42. The ducks were vaccinated with duck hepatitis virus vaccine on day 1, duck plague virus vaccine on day 7, and H5+H7 avian influenza vaccine on day 14. The management protocol and vaccination program were set according to the Technical Regulations of Health Cultivation in Meat Duckling (DB 32/T2692–2014, China) with reference to the Cherry Valley Duck Management Guide.

On day 43, twelve ducks per group (*i.e*., 2 ducks per replicate) were randomly selected, which was followed by euthanizing by CO_2_ asphyxiation. After that, the lengths and weights of the duodenum, jejunum, ileum and cecum were measured. A tissue sample of approximately 2 cm was collected from the middle portion of each intestinal segment. Half of the sample was placed in a 4 % paraformaldehyde solution for later histomorphological analysis, and the other half was placed in liquid nitrogen for later quantitative polymerase chain reaction (**PCR**) analysis. Subsequently, the contents of each intestinal segment was harvested and stored at −70 °C for 16S rDNA analysis. Later each intestinal segment was opened and rinsed with saline to remove the residual chyme, which was followed by collecting the mucosa on the inner wall of the intestine by gently scraping it off with clean glass slides. The mucosa samples were placed in −70 °C freezer for subsequent analysis.

### 2.2 Histomorphological analysis

The paraffin sections of the intestine were prepared and stained with H&E according to the method described previously (Grosset et al., 2019). The height of intestinal villi and the depth of crypts were measured under a light microscope. For each section, five intact and well-oriented villi with their corresponding crypts were randomly selected and measured, and each group had 12 sections with 2 each replicate. The height of intestinal villi refers to the vertical distance between the base and the apex of the villi on the inner wall of intestinal mucosa, and the crypt depth of intestinal villi refers to the vertical distance between the opening of the crypt (*i.e.* the surface of the intestinal mucosa) and the base of the crypt.

### 2.3 Determination of antioxidant capacity of intestinal mucosa

After the mucosa samples were thawed, approximately 2.0 g of tissue was weighed and mixed with 1.8 mL of saline, followed by homogenizing at 4 °C for 2–3 cycles of 10–15 s each using an electric tissue homogenizer. The homogenate was then centrifuged at 16,000 g at 4 °C for 5 min. The supernatant was used for the determination of antioxidant capacity in the intestinal mucosa. The relevant antioxidents determination kits were purchased from Jiancheng Institute of Biological Engineering (Nanjing, Jiangsu, China). The assays were performed according to the manufacturer’s guidelines. .

### 2.4 Quantitative PCR (qPCR) analysis

The method for RNA isolation using the Trizol kit (CAT#DP424; Tiangen Biotechnology (Beijing) Co., Ltd., Beijing, China) was performed as previously described (Vomelová et al., 2009). Briefly, a small amount of intestinal tissue sample (approximately 0.5 g) was placed in a tube containing 1 mL Trizol and homogenized on ice for 2–3 cycles (10–15 s each) using an electric tissue homogenizer, followed by 5 min at room temperature and centrifuging at 16,000 g at 4 °C for 5 min. The supernatant was transferred into a new microtube, and 0.2 mL of chloroform was mixed with the supernatant by shaking the microtube for 30 s. After 10 min at room temperature, the mixture was centrifuged at 16,000 g for 5 min. The supernatant was removed and the white RNA pellet at the bottom of the tube was washed with 75 % ethanol, followed by centrifuging at 16,000 g for 5 min. The procedures from discarding the supernatant to centrifuging were repeated twice. The precipitate was air-dried for 5 min, and dissolved in RNAase-free water. Gel electrophoresis was performed to check the integrity of RNA, followed by using the NanoDrop instrument to determine the concentration and purity (based on optical density **(OD)** values and the ratio at 260 and 280 nm) of RNA samples.

After qualitative and quantitative analysis of the RNA samples, the total RNA concentration was diluted to 300 ng/μL. The reverse transcription reaction was performed with the HiscriptTM Q RT Supermix Reverse Transcription Kit bought from Vazyme Biotech Co., Ltd. (Cat No R123–01, Nanjing, China) according to the manufacturer's instructions. In brief, a solution containing 2 μL of RNA, 4 μL of 4 × genomic DNA wiper and 10 μL of RNase-free H2O was added in a centrifuge tube, mixed and placed in a PCR instrument for 2 min at 42 °C. After removing genomic DNA, 4 μL of 5 × qRT SuperMix Ⅱ was added, followed by performing a reaction at 50 °C for 15 min to synthesize cDNA.

For qPCR, the SYBR Green assay was performed with the Vazyme ACEQ QPCR SYBR Green Master Mix Kit (Vazyme Biotech Co., Ltd., Nanjing, China) as described previously (Hawkins & Guest, 2017). Briefly, the synthesized cDNA was mixed with the reagents in the kit in an appropriate proportion, followed by placing the mixture in a quantitative PCR thermocycler. The reaction was subsequently carried out using the following conditions: pre-denaturation at 95 °C for 5 min, followed by 40 cycles of denaturation at 95 °C for 1 min, and annealing and extension at 60 °C for 30 s. Finally, the relative mRNA expression of the targeted gene was calculated using the 2^-ΔΔCt^ method. There are three technical replicates for each sample. The internal control gene was glyceraldehyde-3-phosphate dehydrogenase (*GAPDH*). The primers for each gene were picked with Primer 3.0 software using the reference sequences in GenBank. The primer sequences are shown in Table 2.

**Table 2:** The list of primer sequences.

Gene	Primer sequences (5′→3′)
*MCP-1*	F:CACCTACGCAAGCAACTGTC
	R:ACGTAGGTGAAACAGCAGGA
*TNF-α*	F:GACAGCCTATGCCAACAA
	R:TCTGAACTGGGCGGTCATA
Claudin-1	F:GTTGCCTTCTAGCAAGCTCT
	R:AGGAGTAAAGACGGGGTCAA
Occludin	F:TTTACGGGAACAAAGAGGCG
	R:GCGCATCCTCGATGTAGTAG
*ZO-1*	F:CACTGTGACCCCAAAACCTG
	R:CTGAGACACAGTTTGCTCCA
*TLR-4*	F:GGGCTACAGGTCAACAGACT
	R:CGTTCACCAGCCGAATACTG
*MYD88*	F:GGTGGTCGTCATTTCAGACG
	R:AACCGCAGGATACTTGGGAA
*GAPDH*	F:GGTTGTCTCCTGCGACTTCA
	R:TCCTTGGATGCCATGTGGAC

### 2.5 Statistical analysis

For statistical verifications, only one-way analysis of variance (**ANOVA**) was carried out with SPSS version 25.0 software, and differences between groups were determined using Duncan's method. The criterion for the significance of difference was set at *P*< 0.05. Results are presented as mean ± standard deviation (**SD**).

## 3 Result

### 3.1 Effect of supplementing lignocellulose on the growth of meat ducks

The results indicated that there was no significant difference in hatch weight between the CON group and the treatment groups (LC1 and LC2 groups). After one week of feeding (Table 3), the average daily gain (**ADG**) was significantly lower in the LC1 and LC2 groups than in the CON group (*P* < 0.05), but the average daily feed intake (**ADFI**) and feed-to-gain ratio (**F:G**) were not significantly different among the groups. The reason for the reduced ADG in lignocellulose-supplemented groups may have been associated with the initial physical adaptation to dietary lignocellulose, which could temporarily reduce nutrient utilization and digestion efficiency.

**Table 3:** Effects of lignocellulose supplementation on growth performance.

Group		CON	LC1	LC2	*P* value
1–7 d	ADG (g)	29.48±1.45^a^	26.77±0.98^b^	26.35±1.76^b^	<0.05
	ADFI (g)	31.98±1.27	30.75±1.25	30.44±1.62	0.16
	F:G	1.09±0.07	1.15±0.05	1.16±0.07	0.18
8–14 d	ADG (g)	59.06±1.71	59.15±1.62	59.07±3.18	1.00
	ADFI (g)	90.93±4.43	88.36±1.94	90.48±1.81	0.31
	F:G	1.54±0.07	1.49±0.05	1.54±0.10	0.44
15–21 d	ADG (g)	51.65±5.00^b^	51.86±3.50^b^	59.29±3.16^a^	<0.05
	ADFI (g)	125.46±6.91^ab^	120.11±5.05^b^	131.02±3.16^a^	<0.05
	F:G	2.43±0.15^a^	2.32±0.20^ab^	2.21±0.12^b^	<0.05
22–28 d	ADG (g)	71.11±5.98^b^	81.50±2.82^a^	80.30±8.28^a^	<0.05
	ADFI (g)	138.34±14.4	150.89±4.38	138.22±8.99	0.07
	F:G	1.95±0.15^a^	1.85±0.07^ab^	1.73±0.15^b^	<0.05
29–35 d	ADG (g)	78.78±8.74	88.88±9.21	84.82±12.32	0.26
	ADFI (g)	228.72±24.79	226.68±6.66	214.73±11.98	0.31
	F:G	2.93±0.42	2.57±0.22	2.56±0.27	0.10
36–42 d	ADG (g)	93.57±12.30	95.27±11.44	87.90±7.94	0.48
	ADFI (g)	265.49±11.17^a^	262.21±8.65^a^	234.66±17.56^b^	<0.05
	F:G	2.89±0.44	2.78±0.24	2.68±0.24	0.55
1–21 d	ADG (g)	46.73±3.16	45.93±2.30	48.24±2.78	0.37
	ADFI (g)	82.79±1.96^a^	79.74±1.31^b^	83.98±0.94^a^	<0.05
	F:G	1.69±0.10	1.65±0.12	1.64±0.10	0.72
22–42 d	ADG (g)	81.15±9.37	88.55±8.63	84.34±9.72	0.40
	ADFI (g)	210.85±17.76	213.26±6.79	195.87±13.31	0.08
	F:G	2.59±0.36	2.40±0.19	2.32±0.23	0.24
1–42 d	ADG (g)	63.94±0.54^b^	67.24±2.16^a^	66.29±3.58^ab^	<0.05
	ADFI (g)	142.90±7.59	147.19±2.87	141.86±5.22	0.25
	F:G	2.24±0.12	2.19±0.05	2.14±0.07	0.17
1 d	BW (g)	58.14±1.49	57.48±1.25	58.56±2.69	0.63
21 d	BW (g)	1039.46±32.95^ab^	1021.93±33.43^b^	1071.53±22.82^a^	<0.05
42 d	BW (g)	2743.63±110.77	2881.48±99.22	2842.62±114.75	0.11

Note: the CON group was fed the basal diet from 1–42 d; the LC1 group was fed the diet supplemented with 0.6 % lignocellulose from 1–42 d; the LC2 group was fed the diet supplemented with 0.3 % lignocellulose from 1–21 d and 0.6 % lignocellulose from 22–42 d ADG denotes the average daily gain, ADFI denotes the average daily feed intake, F:G denotes the feed-to-gain ratio, BW: denotes body weight. The data are presented as mean ± SD. The p-value is for the F-test, *n* = 6.

In the second week (8–14 d), no significant differences were observed in ADG, ADFI, and F:G among the groups. During the third week of feeding (15–21 d), the LC2 group exhibited a significantly higher ADG than the CON and LC1 groups (*P* < 0.05), consistent with the body weight at 21 day of age. This is accompanied by a higher ADFI (*P* < 0.05) and a lower F:G (*P* < 0.05) in the LC2 group than the LC1 group. It is noteworthy that compared to the second week (8–14 d), the ADG of each group was decreased or remained unchanged, and the F:G ratio was increased during the third week (15–21 d), likely due to stress associated with vaccination and relocation to a new facility.

In the fourth week (22–28 d), ADG significantly increased in both LC1 and LC2 groups compared with the CON group (*P* < 0.05), while F:G decreased significantly (*P* < 0.05). During the fifth week (29–35 d), no significant differences in ADG, ADFI, and F:G among treatments were observed. In the terminal week (36–42 d), ADG and F:G remained statistically similar among groups, although ADFI was significantly lower in the LC2 group than in the CON and LC1 groups (*P* < 0.05).

Over the entire experimental period (1–42 d), lignocellulose supplementation had no significant effect on ADFI and F:G. However, the LC1 group showed a significantly higher ADG than the CON group (*P* < 0.05), while the LC2 group exhibited a numerically higher but statistically comparable ADG. These results suggest that moderate levels of dietary lignocellulose may enhance long-term growth performance without compromising feed efficiency in Cherry Valley ducks.

### 3.2 Effect of supplementing lignocellulose on intestinal development of meat ducks

Experimental data revealed structural differences in the absolute length of different intestinal segments. The duodenal length of LC1 and LC2 groups was significantly longer than that of the CON group (*P* < 0.05) (Table 4), whereas the jejunal length of the LC1 group was significantly shorter than that of the CON and LC2 groups (*P* < 0.05). The ileum of the LC2 group was significantly longer than that of the CON and LC1 groups (*P* < 0.05). In addition, the cecal length of LC1 group was significantly longer than that of the LC2 group (*P* < 0.05).

**Table 4:** Effects of supplementing lignocellulose on intestinal development.

Group	CON	LC1	LC2	*P* value
Absolute length (cm)				
Duodenum	35.04±1.52^b^	36.58±0.29^a^	37.17±0.42^a^	<0.05
Jejunum	95.71±3.36^a^	91.92±0.69^b^	94.33±1.74^ab^	<0.05
Ileum	94.83±3.48^c^	101.29±0.76^b^	106.21±1.45^a^	<0.05
Cecum	40.00±0.42^ab^	40.75±0.91^a^	39.42±0.51^b^	<0.05
Absolute weight (g)				
Duodenum	10.42±0.27	10.21±0.20	10.14±0.39	0.27
Jejunum	28.46±0.98^a^	25.67±0.34^c^	26.88±1.30^b^	<0.05
Ileum	30.54±1.37^b^	33.92±0.54^a^	33.46±3.21^a^	<0.05
Cecum	4.96±0.10^b^	5.75±0.22^a^	5.75±0.54^a^	<0.05
Relative weight (g/kg)				
Duodenum	3.80±0.18^a^	3.54±0.14^b^	3.57±0.20^b^	<0.05
Jejunum	10.37±0.55^a^	8.91±0.33^c^	9.46±0.60^b^	<0.05
Ileum	11.13±0.67	11.77±0.45	11.77±1.22	0.35
Cecum	1.81±0.08^b^	2.00±0.10^a^	2.02±0.21^a^	<0.05

Note: the CON group was fed the basal diet from 1–42 d; the LC1 group was fed the diet supplemented with 0.6 % lignocellulose from 1–42 d; the LC2 group was fed the diet supplemented with 0.3 % lignocellulose from 1–21 d and 0.6 % lignocellulose from 22–42 d The data are presented as mean ± SD. The p-value is for the F-test, *n* = 6.

Moreover, the data on the absolute weight of different intestinal parts showed that the duodenal weight did not alter significantly among the groups (Table 4). The jejunal weight in the CON group was significantly higher than that in the LC1 and LC2 groups (*P* < 0.05). The ileal and cecal weights in both the LC1 and LC2 groups were significantly higher than that in the CON group (*P* < 0.05). In terms of the relative weight of different intestinal parts, both the duodenal and jejunal weights in LC1 and LC2 were significantly lower than the CON group (*P* < 0.05), but the cecal weight in LC1 and LC2 was significantly higher than the CON group (*P* < 0.05) (Table 4). Except for the significant increase in relative jejunum weight in the LC2 group compared to the LC1 group (*P* < 0.05), no significant differences were observed between LC1 and LC2.

These findings indicated that lignocellulose supplementation, particularly at higher levels, promoted elongation of the small intestinal segments especially the duodenum and ileum parts and increased the mass of the distal intestine, suggesting enhanced intestinal development and dietary fiber adaptation.

The data on intestinal morphology showed that duodenum (Table 5) crypt depth in both the LC1 and LC2 groups was significantly smaller than that in the CON group (*P* < 0.05), whereas in the jejunum segment, the three indices of intestinal structure (the crypt depth, the villus height and their ratio) were not significantly different between the groups. In the ileum, the villus height in the LC1 and LC2 groups was significantly greater than that in the CON group (*P* < 0.05), but the ratio of villus height to crypt depth in the LC1 group was significantly higher than that in the CON and LC2 groups (*P* < 0.05).

**Table 5:** Effects of supplementing lignocellulose on intestinal morphology.

Group	CON	LC1	LC2	*P* value
Duodenum				
Villus height (μm)	1092.22±153.00	1133.48±89.77	1127.72±133.96	0.84
Crypt depth (μm)	245.48±18.64^a^	204.53±20.16^b^	200.12±35.69^b^	<0.05
V/C	4.48±0.81	5.66±0.88	6.00±1.52	0.08
Jejunum				
Villus height (μm)	888.35±165.56	981.44±155.00	1031.28±163.04	0.32
Crypt depth (μm)	199.02±57.00	182.90±40.51	197.78±56.95	0.84
V/C	4.59±0.61	5.45±0.51	5.87±2.13	0.26
Ileum				
Villus height (μm)	683.96±123.01^b^	803.61±53.84^a^	810.72±91.29^a^	<0.05
Crypt depth (μm)	165.03±51.19	136.69±27.68	151.68±27.90	0.42
V/C	4.47±1.42^b^	6.14±1.03^a^	5.51±1.03^ab^	<0.05

Note: the CON group was fed the basal diet from 1–42 d; the LC1 group was fed the diet supplemented with 0.6 % lignocellulose from 1–42 d; the LC2 group was fed the diet supplemented with 0.3 % lignocellulose from 1–21 d and 0.6 % lignocellulose from 22–42 d “Ratio” denotes “Ratio of Villus hight to crypt depth”. The data are presented as mean ± SD. The p-value is for the F-test, *n* = 6.

### 3.3 Effects of supplementing lignocellulose on the antioxidant activity of the intestinal mucosa

In the jejunal mucosa (Table 6), the total antioxidant capacity (U/mg prot) of LC1 and LC2 groups was significantly higher than that of CON group (*P* < 0.05). This study revealed that the malondialdehyde content (nmol/mg prot) of jejunal mucosa of the LC1 group was significantly lower than that of the LC2 group (*P* < 0.05), and the latter was also significantly lower than that of the CON group (*P* < 0.05).

**Table 6:** Effects of supplementing lignocellulose on the antioxidative capacity of jejunal and ileal mucosa.

Group	CON	LC1	LC2	*P* value
Jejunum				
Total superoxide dismutase activity (U/mg prot)	47.00±4.21	48.19±4.36	48.59±5.19	0.83
Glutathione (mg/g prot)	24.69±0.69	30.43±2.06	25.02±5.24	<0.05
Glutathione S-transferase (U/mg prot)	48.79±7.42	45.39±7.54	47.36±12.42	0.82
Total antioxidant capacity (U/mg prot)	3.17±0.07^b^	3.69±0.42^a^	3.86±0.49^a^	<0.05
Malondialdehyde (nmol/mg prot)	1.39±0.32^a^	0.60±0.15^c^	1.04±0.24^b^	<0.05
Ileum				
Total superoxide dismutase activity (U/mg prot)	52.08±11.83	41.54±6.81	47.28±9.21	0.19
Glutathione (mg/g prot)	11.06±2.84	10.53±2.40	11.54±2.47	0.80
Glutathione S-transferase (U/mg prot)	41.41±15.65^b^	40.85±10.39^b^	62.74±13.67^a^	<0.05
Total antioxidant capacity (U/mg prot)	0.79±0.07^b^	1.10±0.07^a^	1.10±0.04^a^	<0.05
Malondialdehyde (nmol/mg prot)	0.75±0.20	0.55±0.15	0.62±0.17	0.16

Note: the CON group was fed the basal diet from 1–42 d; the LC1 group was fed the diet supplemented with 0.6 % lignocellulose from 1–42 d; the LC2 group was fed the diet supplemented with 0.3 % lignocellulose from 1–21 d and 0.6 % lignocellulose from 22–42 d The data are presented as mean ± SD. The p-value is for the F-test, *n* = 6.

Meanwhile, glutathione S-transferase (U/mg prot) activity in ileal mucosa of the LC2 group was significantly higher than that of the CON and LC1 groups (*P* < 0.05); and the total antioxidant capacity (U/mg prot) in ileal mucosa of the LC1 and LC2 groups was significantly higher than that of the CON group (*P* < 0.05) (Table 6).

### 3.4 Effects of supplementing lignocellulose on the expression of genes related to tight junction, immunity and inflammation in the intestine

**Fig. 1:**
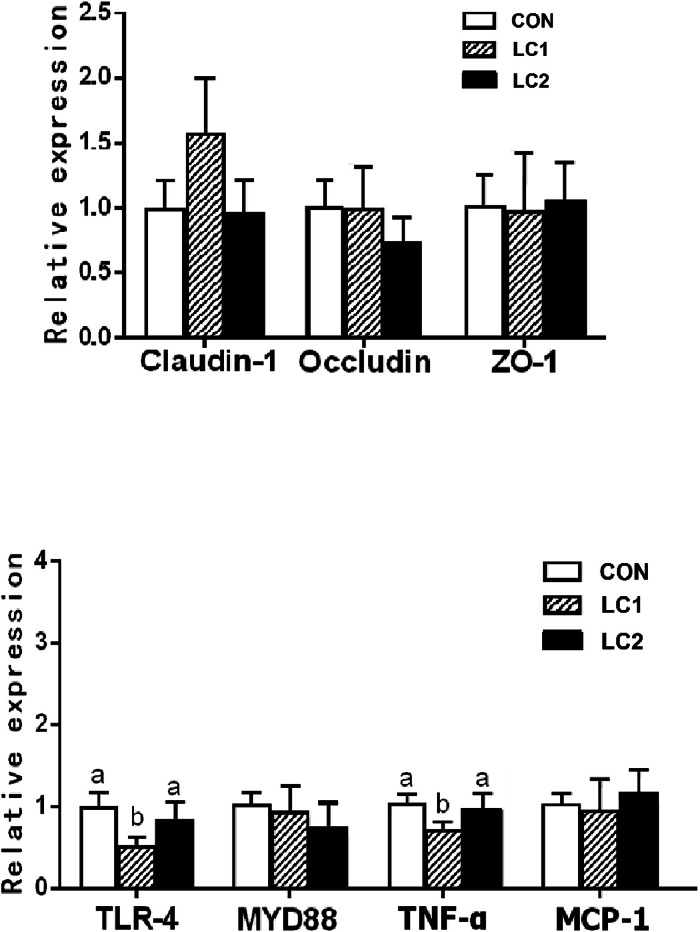
Effects of lignocellulose supplementation on the mRNA expression of Claudin-1, Occludin, *ZO-1* (A), *TLR-4, MYD-88*, T*NF-α* and *MCP-1* (B) in ileum. Note: the CON group was fed the basal diet from 1–42 d; the LC1 group was fed the diet supplemented with 0.6 % lignocellulose from 1–42 d; the LC2 group was fed the diet supplemented with 0.3 % lignocellulose from 1–21 d and 0.6 % lignocellulose from 22–42 d The p-value is for the F-test, *n* = 6.

The mRNA expression of genes related to tight junction (Claudin-1, Occludin, and *ZO-1*) and immunity or inflammation (*TLR-4, MYD88, TNF-α* and *MCP-1*) in duck intestinal tissues were determined by qPCR, and the data revealed that supplementing lignocellulose did not significantly alter the mRNA expression levels of tight junction proteins (Claudin-1, Occludin, and *ZO-1*) in ileal tissues (Fig. 1A), nor did it significantly affect the expression of immune/inflammation related genes (*TLR-4, MYD88, TNF-α* and *MCP-1*) (Fig. 1B).

## 4 Discussion

Growth performance is one of the most economically important traits in livestock and poultry production. Although previous studies have reported that crude fiber content in feeds acts as an anti-nutritional factor and feed diluent that can reduce feed digestibility and growth performance in poultry (Rougière & Carré, 2010), recent evidence indicates that appropriate amounts of crude fiber do not necessarily impair growth performance of poultry, and even enhance the intestinal health and promote animal growth (Jha & Mishra, 2021). Compared to other livestock and poultry, there are only a few studies on the supplementation of crude fiber to meat duck diets. For example, a study on the effects of different crude fiber levels (2.4 %, 3.8 %, 5.3 %, 6.7 %) from soybean hulls on Pekin ducks revealed that feeding dietary fiber decreased duodenal villus height and mucosal thickness, while significantly increasing villus width (El-Katcha et al., 2021). It also elevated serum high-density lipoprotein levels, enhanced antioxidant capacity, and improved immune function - with no negative effect on body weight. Another study investigated the effects of dietary crude fiber levels (1.46 %, 3.09 %, 4.15 %, 6.18 %, 7.52 % and 9.03 %) on growth performance, gizzard development, intestinal morphology, and nutrient utilization in Cherry Valley meat ducks from 1 to 21 d of age (Han et al., 2017). The results of this study showed that the body weight, body weight gain, feed-to-gain ratio and feed intake at 21 d of age increased with the increase of crude fiber levels from 1.46 % to 9.03 % in the starter diet of ducks, particularly, the starter diet containing 7.52 % crude fiber promoted gizzard development and improved jejunal morphology.

These findings indicate that meat ducks from 1 to 21 d can adapt to a wide range of dietary fiber levels. The results of the present study also agree with previous findings on the growth-promoting and antioxidant-enhancing effects of dietary fiber.

The current study showed that the ADG of meat ducks in the LC1 group from 1–42 d was significantly higher than that of the CON group, indicating that supplementation with 0.6 % lignocellulose improved growth performance.Weekly ADG patterns showed that both LC1 and LC2 ducks exhibited significantly lower ADG in the first week compared with the CON group. Supplementation of lignocellulose was detrimental to the growth of ducklings in the first week, probably due to reduced palatability and requirement of gut adaptation, the ducklings began to adapt to the supplemented lignocellulose in the second week, which resulted in no significant difference in the growth between the groups. In the third week, the ADG of the LC2 group was significantly higher than that of the CON and LC1 groups, whereas LC1 did not differ from CON, suggesting that the growth-promoting effect of lignocellulose is dose-dependent. In the fourth week, the ADG of both LC1 and LC2 groups was significantly higher than that of the CON group. This suggests that the growth-promoting effect of high-dose lignocellulose requires a longer adaptation period and may depend on the physiological stage of the ducks. In the fifth week, although the ADG of the LC1 group was not significantly higher than that of the CON group, the difference between the groups was similar to that in the fourth week, suggesting that the growth-promoting effect of high-dose lignocellulose was still in progress. However, the difference in ADG between the LC2 group and the CON group was no longer significant, suggesting that the ducks needed a period of time to adapt to the increased dose of lignocellulose at a later stage.

The ADG of the CON group and the LC1 group did not differ substantially in the sixth week, indicating that the growth-promoting impact of high-dose lignocellulose had reached to a physiological equilibrium. The LC2 group, on the other hand, had a slightly lower ADG than the CON group, suggesting that the stimulatory impact of lignocellulose decreases with age and that a greater lignocellulose content at later growth stages may necessitate a longer adaptation period. Overall, these findings show that the growth-promoting effects of lignocellulose are dependent on both the age of ducks and the degree of its incorporation.

Considering that meat ducks require time to adapt to the change in lignocellulose dose and that the production cycle before marketing is short, frequent dose adjustments may not be appropriate. A constant 0.3 % supplementation level may be more effective than 0.6 %, though further validation is needed.

A study conducted on Carlos geese also revealed that supplementing 8 % high dietary fiber can significantly promote the development and growth of the intestinal tract (Zhou et al., 2018). Dietary crude fiber at an optimum range can promote the development of gizzard and increase the thickness of the intestinal muscle layer in meat ducks (Han et al., 2016). Crude fiber supplementation in the feed of meat ducks can increase the villus height of the ileum at 7 d of age and decrease the crypt depth of the ileum at 21 d and 35 d of age (Han et al., 2016). As the increase of villus height indicates the increase of villus absorption ability, the decrease of crypt depth indicates the maturation rate and secretion capacity of intestinal cells, a higher villus-to-crypt ratio supports a greater absorptive surface area and improved nutrient uptake. Thus, the growth-promoting effects of lignocellulose observed in this study may also be partly attributed to the improvement of the morphological structure of the intestine by lignocellulose.

It is known that the integrity of the intestinal wall is essential for the maintenance of intestinal function. Oxidative stress causes an inflammatory response that results in cell death and tissue damage (John et al., 2011). Han also reported that dietary fiber supplementation enhances serum antioxidant capacity in meat ducks. Consistent with this, the present study also indicates that lignocellulose supplementation improved the antioxidant capacity of the jejunal and ileal mucosa. Enhanced intestinal antioxidant activity helps maintain mucosal barrier integrity.

However, lignocellulose did not significantly affect the mRNA expression of claudin-1, occludin, and ZO-1, the genes involved in intestinal tight junction. These proteins are essential structural components that regulate cell–cell adhesion and maintain intestinal barrier function (Turner, 2008). A reduction in their expression can increase intestinal permeability, making the host more susceptible to pathogens (Shen, 2009).

Moreover, lignocellulose supplementation did not alter the expression of immune or inflammation-related genes (*TLR-4, MYD88, TNF-α*, and *MCP-1*). TLR-4 activates the NF-κB signaling pathway through MYD88, inducing pro-inflammatory cytokines such as TNF-α and MCP-1 (Kawai & Akira, 2009; Chen et al., 2018). The absence of changes in these genes indicates that lignocellulose did not trigger intestinal inflammation.

This lack of inflammatory response may be attributed to the enhanced antioxidant capacity of the intestinal tissue, which can suppress oxidative stress and modulate inflammation-related signaling pathways.

Finally, for most parameters, differences between the two supplementation schedules (LC1 and LC2) were less pronounced than differences between either treatment and the CON group. In terms of ADG, ADFI, and body weight, the ADG and ADFI of the LC2 group at week 3 were significantly higher than those of the LC1 group, but the situation of ADFI was reversed at week 6. This may be due to the fact that compared to the addition of 0.3 % lignocellulose, 0.6 % significantly reduced the palatability, digestion and absorption capacity of the feed, requiring a longer adaptation period. The reversal in ADFI at the sixth week of age may be related to the LC1 group adapting to 0.3 % lignocellulose easier than 0.6 % lignocellulose and the switch from 0.3 % to 0.6 % lignocellulose in the LC2 group at 21 days of age. In terms of intestinal length and weight, at 6 weeks of age, the ileum length and the absolute and relative weights of the jejunum in LC2 were significantly greater than those in LC1, while the cecum length of LC2 was significantly shorter than that of LC1. These differences may be associated with varying degrees of physical stimulation of the intestine by different lignocellulose addition levels, the switch in lignocellulose addition rates in the feed, the physiological characteristics of ducks at different growth stages, the length of the adaptation period, and the regulation of intestinal oxidative stress and inflammatory factor expression levels. Based on the results of this study, continuously feeding a diet with 0.3 % lignocellulose may be more conducive to duck growth.

## 5 Conclusions

The supplementation of lignocellulose to the diet plays its role in promoting the growth of ducks and improving the feed conversion rate of meat ducks by enhancing intestinal structure and function, and enhancing intestinal antioxidant capacity without inducing gene expression involved in intestinal tight junction and immunity/inflammation. Although the supplementation of 0.6 % lignocellulose significantly increased the ADG of ducks during 1–42 d of age, the effect of supplementing 0.3 % lignocellulose at a constant level may be better, but this needs in-depth validation in future.

## Ethical statement

All animal experiments in this study were performed according to the ethical rules and regulations related to animal use and welfare, and the animal experimental protocol was approved by the Institutional Animal Care and Use Committee of Yangzhou University (approval number SYXK(Su)2021–0026).

## Declaration of generative AI and AI-assisted technologies in the writing process

During the writing process of this manuscript, the authors used ChatGPT 4.0 for English editing purposes, including checking for grammatical errors and language polishing.

## Data availability

None of the data or the models were deposited in an official repository. The datasets generated and/or analyzed in the current study are available from the corresponding author upon reasonable request.

## CRediT authorship contribution statement

**Xinzhi Geng:** Writing – original draft, Methodology, Investigation. **Zhenzhen Chen:** Writing – original draft, Investigation. **Jian Wang:** Resources, Funding acquisition. **Biao Dong:** Resources. **Jing Ge:** Methodology, Investigation. **Minmeng Zhao:** Formal analysis. **Long Liu:** Formal analysis. **Daoqing Gong:** Writing – review & editing, Methodology. **Haixia Liu:** Writing – review & editing, Conceptualization. **Tuoyu Geng:** Writing – review & editing, Funding acquisition, Conceptualization.

## Declaration of competing interest

The authors declare that they have no known competing financial interests or personal relationships that could have appeared to influence the work reported in this paper.
